# Protein Crowding Effects on Hydration Water Dynamics

**DOI:** 10.1021/acs.jpclett.4c03391

**Published:** 2025-02-24

**Authors:** Luigi Caminiti, Maria Taddei, Sara Catalini, Paolo Bartolini, Andrea Taschin, Renato Torre

**Affiliations:** † Dipartimento di Fisica ed Astronomia, Università degli Studi di Firenze, Via G. Sansone 1, 50019 Sesto Fiorentino (FI), Italy; ‡ European Laboratory for Non-Linear Spectroscopy, Via Nello Carrara 1, 50019 Sesto Fiorentino (FI), Italy; § Dipartimento di Fisica e Geologia, Università degli Studi di Perugia, Via Alessandro Pascoli, 06123 Perugia (PG), Italy; ∥ Consiglio Nazionale delle Ricerche - Istituto Nazionale di Ottica, Via Nello Carrara 1, 50019 Sesto Fiorentino (FI), Italy

## Abstract

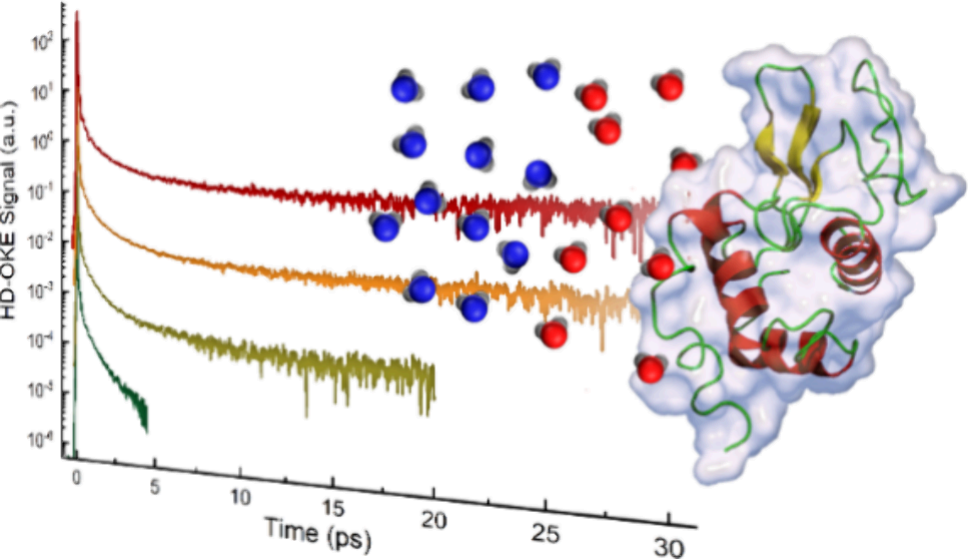

We propose a time-resolved optical Kerr effect study of the structural and vibrational dynamics of the hydration water surrounding the lysozyme on a very fast time scale. Measurements as a function of lysozyme concentration make it possible to distinguish the hydration water contribution from that of both the bulk water and the protein. Our results provide experimental evidence of the existence of two structural dynamics of hydration water, associated with a hydrogen bond exchange relaxation process and with the reorganization of water molecules induced by protein structural fluctuations. Likewise, we evaluated the vibrational dynamics of the water hydration layer at subpicosecond time scales. Our measurements of hydration water properties reveal the presence of a crossover point at a specific protein concentration. This crossover marks the transition between two clustering regimes with distinct hydration characteristics and establishes a possible threshold for protein crowding.

Water molecules that hydrate proteins’ surfaces are fundamental for ensuring their structural flexibility, which is among the key requirements to guarantee their ability to perform physiological functions. Protein mobility is modulated by water dynamics, and protein motions influence the stability of water hydrogen bonds (HBs). However, the exact role of the hydration layers in regulating continuous protein motion is still an object of intense debate.^1−4^ Even the measurement of the real extent of the hydration shell (i.e., the number of water molecules whose properties are influenced by the presence of the protein) is the subject of various scientific considerations due to the strong correlation with the spectroscopic observables investigated.^4−6^

A widely studied system is that of lysozyme/water solutions, which provides an excellent testing ground to better understand the physical principles behind several biological phenomena involving proteins. Indeed, this globular protein, which can be easily isolated from the white of a chicken egg, has a remarkably human-like structure and can easily create amyloid aggregates in vitro. This capability is critical for understanding the molecular pathways that lead to the production of amyloid oligomers and mature amyloid fibrils,^7−9^ which are responsible for many degenerative diseases.^10^ Furthermore, lysozyme is one of the few basic proteins with a high isoelectric point, pH 11, which allows for easy charging of the protein surface by varying the pH or using other parameters such as ionic strength and protein concentration and thus sheds light on the role of the excluded volume effect and the interaction potential in the formation of transient protein clusters.^11−13^

The clustering of proteins in a solution of lysozyme and water is closely intertwined with the phenomenon of metastable liquid–liquid phase separation (LLPS).^14−16^ LLPS leads to the emergence of both a sparse and a densely populated phase; various aggregation structures can quickly emerge with slight alterations in physical parameters like ionic strength, concentration, and temperature. Hydration water plays a crucial role in regulating these condensation phenomena, showing a deep connection between water properties and protein reorganization inside the solution. Condensate formation leads to the release of the so-called hydrophobic water (water molecules hydrating the hydrophobic moieties of the protein) and to the maintenance of the hydrophilic water (water molecules hydrating the hydrophobic moieties) inside the condensate.^17−20^

A variety of experimental techniques have been employed to examine the lysozyme/water solution. However, only a limited number have specifically targeted the measurement of hydration water dynamics due to the inherent challenges in distinguishing these dynamics from the contributions of proteins and solvents to the measured signal. Experimental investigations utilizing optical spectroscopic techniques hold promise in accessing ultrafast dynamics and thus in measuring hydration water dynamics. Specifically, optical spectroscopic studies have been conducted on lysozyme/water solutions, exploring the frequency^21−23^ and time^24−27^ domains. The findings from these experimental investigations offer interesting insight into lysozyme interactions and dynamics. Nevertheless, the collected experimental data do not facilitate a detailed examination of hydration water dynamics, so these peculiar dynamics are still not fully disclosed. The earlier time-resolved experimental investigations^24,25^ focused on protein dynamics and do not offer a detailed analysis of water dynamics. The recent studies both in frequency, Perticaroli et al.,^21,22^ and in time domain, Mazur et al.,^26^ do not reveal the articulated nature of hydration water dynamics; they analyzed only the structural dynamics reproduced by a simple exponential relaxation associated with the breaking and reformation of hydrogen bonds forming the water network. Therefore, all of these studies do not reveal the presence of a second structural dynamic, predicted by simulative work,^28−30^ and do not characterize the intermolecular vibration of hydration water.

In this context, we performed an extensive experimental spectroscopic analysis employing time-resolved optical Kerr effect techniques on lysozyme/water solutions. Our aim was to investigate the rapid dynamics of hydration water and detect any crowding effects caused by different protein concentrations. Our work succeeds in giving detailed information on the structural and vibrational dynamics of hydration water over a very wide range of concentrations. Furthermore, we sought to establish correlations between these observations and the reported occurrence of LLPS in the literature.

Our findings experimentally confirm the presence of two relaxation times associated with the hydrogen bond exchange relaxation process and with the reorganization of water molecules induced by protein structural fluctuations. Furthermore, the analysis of both fast vibrational and slow structural dynamics shows that some observed changes on measured parameters occur at a definite concentration threshold, which appears connected to the occurrence of variations in cluster formation.

The heterodyne-detected optical Kerr effect (HD-OKE) technique is a nonlinear time domain spectroscopy method.^31−35^ In brief, a first nonresonant laser pulse with linear polarization (pump pulse) generates a transient anisotropic modification of the refractive index in an optically transparent and isotropic sample. The response of the material to the perturbation produced by the pump pulse is investigated with a second, circularly polarized pulse (probe pulse). The experimental control of the time delay between these two pulses enables the direct measurement of sample dynamics in the time domain, i.e., the relaxation of the non-equilibrium coherent state, induced by the pump pulse in the molecular ensemble, toward the equilibrium state. Thanks to the femtosecond laser pulses employed, the accessible time window of this technique covers an extended time range, spanning from a few femtoseconds to tens of picoseconds.

The signal measured in the HD-OKE experiment can be expressed as^35^
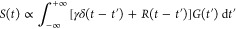
1
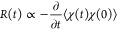
2where *γδ*(*t*) is the instantaneous electronic response, *R*(*t*) is the third-order nuclear response of the sample, and *G*(*t*) is the instrumental function. The latter represents the temporal cross-correlation of the laser pulse intensity. An accurate evaluation of the instrumental function is a critical element of an HD-OKE measurement for a correct extraction of the sample response function.^35,36^ Response function *R*(*t*) is directly related to the time derivative of the correlation function of first-order equilibrium susceptibility χ(*t*) (see eq 2). χ(*t*) is defined by the dynamics of the entire molecular system investigated and includes many intra- and intermolecular motions, so its expression can be very complex and hardly can be described by a rigorous molecular model. Nevertheless, in the case of protein/water solutions, a general separation of the dynamic time scales is present;^37^ on the fast subpicosecond time scale, the dynamics are mainly due to the vibrational dynamics of intramolecular modes of protein and intermolecular H-bond modes of water, while beyond the picosecond time scale, the dynamics are prevalently associated with the overall water structural relaxations. The dynamics of the protein structural transitions and rotational diffusion are very slow and are not accessible in the time window probed in the present experiment.

The Fourier transform of the HD-OKE response function, *R*(ω), is directly connected with the spectrum measured in a depolarized light scattering (DLS) experiment. The imaginary part of *R*(ω), Im[*R*(ω)], is obtained by Fourier transform of the measured HD-OKE signals after the deconvolution processes from the instrumental function Im[*R*(ω)] = Im[*S*(ω)/*G*(ω)].

All of the experimental measurements were acquired and analyzed in the time domain. Figure 1A reports, on a semilog scale, the OKE signal of some investigated lysozyme concentrations, while Figure 1B shows the same data in the Fourier transformed domain. To perform a compared data analysis of the vibrational and structural contributions of all of the samples, a specific normalization procedure is needed. The normalization accounts for the signal intensity change due to the different concentrations of lysozyme in solution. This procedure is described in section S7 of the Supporting Information. Figure 1B shows the normalized response function spectra of some of the recorded data. The lysozyme concentration increase causes an increase in the signal intensity and a change in the spectral shape due to the structural and vibrational contributions.

**Figure 1:**
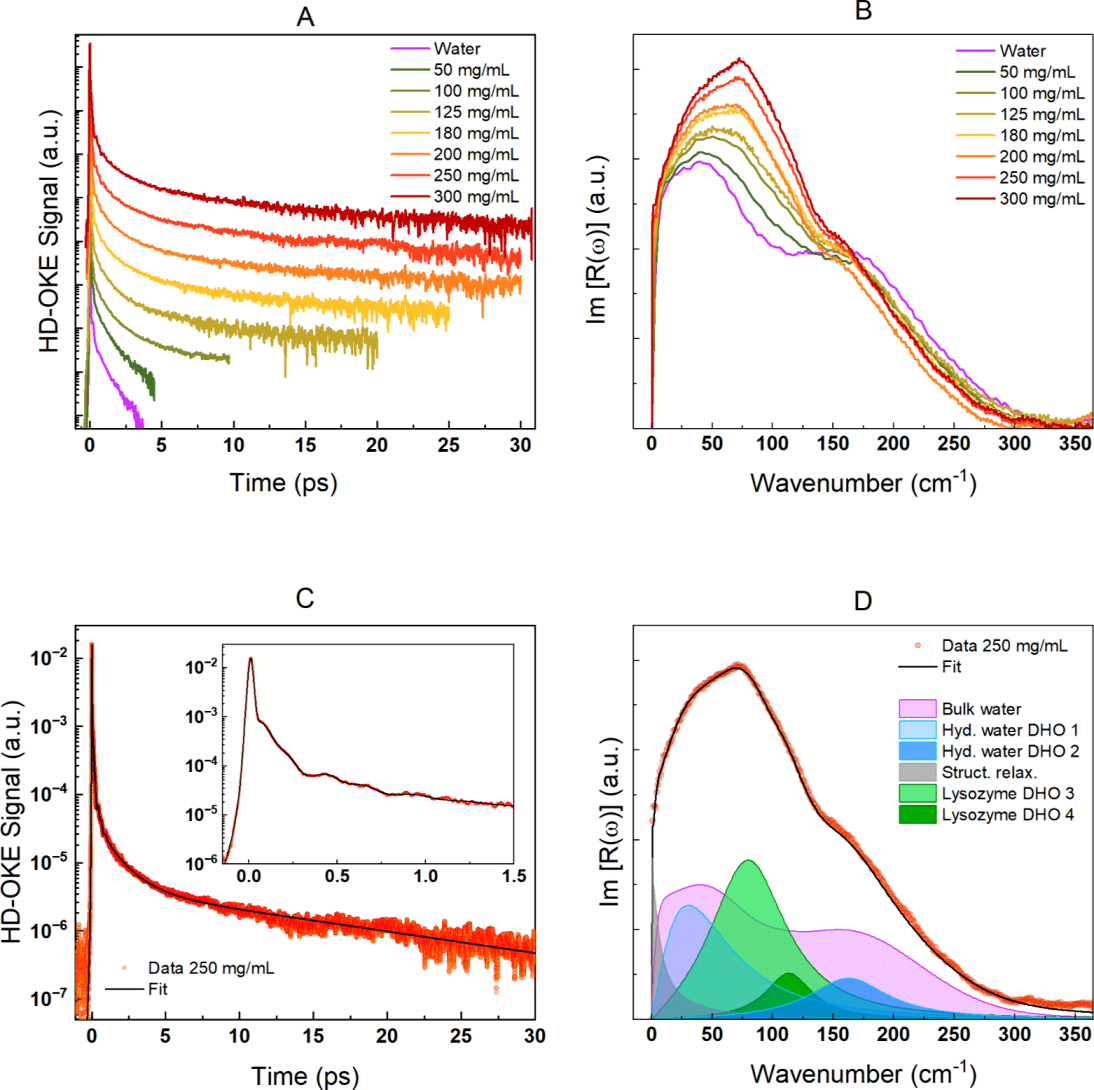
(A) HD-OKE experimental data of 50, 100, 125, 180, 200, 250, and 300 mg/mL (from green to red) lysozyme solutions, compared with that of pure water (purple trace). Each curve is presented multiplied by an appropriate factor to avoid overlapping. (B) Response function spectra of some of the kinetics of panel A after the deconvolution from the instrumental response and the normalization procedure described in the Supporting Information. (C) Semilog experimental signal of the 250 mg/mL (red points) lysozyme solution and its time fitting function (black line). The inset shows signal oscillations due to vibrational dynamics in the short time range (<2 ps). (D) Spectrum of the response function of a 250 mg/mL lysozyme solution. The experimental data (red points) and the relative fitting curve (black line) are compared with the bulk water contribution (purple shaded curve) imposed on the fitting function (*F*_bulk_*R*_bulk_) and with the other spectral contributions. In detail, a biexponential relaxation (gray shaded curve) and two damped harmonic oscillators associated with the hydration water response (shaded blue curves) and two other damped harmonic oscillators associated with the protein response (shaded green curves), all extrapolated from the fitting function (*R*_h-ly_).

In Figure 1C, we report HD-OKE signal kinetics and its fitting function for the measurement at a 250 mg/mL protein concentration. The experimental signal of this sample (red points) shows a long structural exponential decay of tens of picoseconds, while the inset in the short time range (<2 ps) shows the oscillations due to the intermolecular vibrational dynamics in a solution of both protein and water.

To fit the measured signal by a relatively simple function, we utilized a phenomenological model based on the multi-damped harmonic oscillators (m-DHOs) approach^36^ (see section S2 of the Supporting Information). It should be noted that unlike the majority of OKE measurements in the literature,^25,26^ we performed all of the data fitting in the time domain. The fitting process enables disentangling the different contributions coming from bulk water, hydration water, and protein. The HD-OKE response function is composed by the bulk water response, *R*_bulk_(*t*), and by the hydration water and lysozyme response function, *R*_h-ly_(*t*), so that
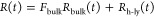
3

Bulk water response *R*_bulk_(*t*) is simulated by an analytical function that is fixed and defined by the independent data analysis of the pure water OKE signal;^35^ this response is weighted by the bulk water volume fraction, *F*_bulk_, present in each lysozyme sample considered. This fraction parameter was calculated following the estimate made by Camisasca et al.,^29^ where the hydration water corresponds to the first hydration layer around each lysozyme molecule (see section S6 of the Supporting Information). Molecular simulation^38^ studies show the perturbation induced by the protein decreases rapidly with the distance from the surface, and thus, it is reasonable consider only the first hydration shell as hydration water. In section S5 of the Supporting Information, we show that performing the data analysis considering two layers instead of one gives the same results with only a rescaling of the extracted parameters.

*R*_h-ly_(*t*) is the response function related to protein and hydration water contributions. This includes two components: a structural response, *R*_struct_(*t*), and a vibrational response, *R*_osc_(*t*):
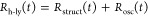
4

According to our data analysis, the structural response can be quite well reproduced by a biexponential decay, characterized by two structural relaxation times, τ_1_ and τ_2_ (see section S3.2 of the Supporting Information). The vibrational response can be simulated by a series of damped harmonic oscillators (DHOs), and it requires a minimum of four DHOs.

Through our rigorous data analysis, we have concluded that these are the minimum number of functions that align with the physical model and are necessary to adequately reproduce the main dynamic features of the HD-OKE data (section S3.3 of the Supporting Information).

The errors associated with the fitting parameters are primarily influenced by the reproducibility of the experimental conditions and measurements. As a result, these errors are estimated by fitting a series of measurements, and they are determined by comparing the dispersion of values from numerous measurements of the same sample.

Figure 1D shows the data of the 250 mg/mL lysozyme solution in the frequency domain (red points) and the Fourier transform of the fit (black line), which was performed in the time domain. In addition, Figure 1D shows all of the components contributing to the fit. These are the *R*_bulk_(*t*) function, weighted by the *F*_bulk_ factor, and the *R*_h-ly_(*t*) function, composed of the biexponential contribution (gray shaded curve) and the four damped harmonic oscillators (blue and green shaded curves). In the following section, we provide a deeper description of these dynamic contributions present in the HD-OKE signal.

Here we analyze the relaxation dynamics contained in the *R*_struct_(*t*) fitting function, i.e., the biexponential decay, characterized by two structural relaxation times, τ_1_ and τ_2_.

These relaxation dynamics are limited to the time range of tens of picoseconds and can be addressed only to the hydration water dynamics since the protein relaxations, both structural and rotational, are characterized by much slower time scales.^37^ The characterization of these hydration water relaxation times is not affected by the data analysis, whatever it may be, and whatever bulk water volume fraction is considered.

Our HD-OKE data reveal that hydration water dynamics is characterized by two separate relaxation processes, each operating on distinct time scales: one occurring within a few picoseconds and the other spanning tens of picoseconds. This observation aligns with the predictions of computer simulation studies of the lysozyme/water system^28,29^ and findings from time-resolved fluorescence spectroscopic studies of protein solvation in other protein/water solutions.^39,40^

According to these prior investigations, these two relaxation processes are related to two distinct dynamical phenomena:

- The rapid relaxation, observed in our data to be approximately 1.6 ps, is driven by the breaking and reforming of hydrogen bond networks associated with OH large-amplitude jumps.^4,28^ This phenomenon mirrors the α-relaxation process in the bulk water^28,29^ but is slowed by a factor of ∼3 due to the binding of water molecules to both hydrophilic and hydrophobic protein residues.^21,29,30^
- The slower relaxation, measured in a range from ∼10 to ∼20 ps, is linked to a structural dynamical phenomenon influenced by rearrangement dynamics driven by the structural fluctuations of the protein. This dynamic is absent in bulk water.

Figure 2 reports the values of the relaxation times obtained by the biexponential fit, τ_1_ and τ_2_, of the experimental data at variable concentrations. Both relaxation times remain almost constant as the concentration changes within the experimental errors; thus, these two dynamics appear to be weakly unaffected by the increasing lysozyme concentration in solution. Indeed, the protein aggregation phenomena are regulated by protein–protein interactions and protein–water interactions. The balance among short-range potential attraction, long-range electrostatic repulsion, and water hydrophobic/hydrophilic interactions leads to the formation of transient protein clusters in dynamic equilibrium with each other and with the isolated proteins in the solution. Clusters are present even at low lysozyme concentrations, but above a certain concentration, cluster formation is energetically favored over the condition of monodisperse protein solutions.^11,13,22,41,42^ Above this concentration, the clustering processes intensify; a further increase in concentration promotes an increase in the number of existing clusters and/or the formation of more extensive clusters.

**Figure 2:**
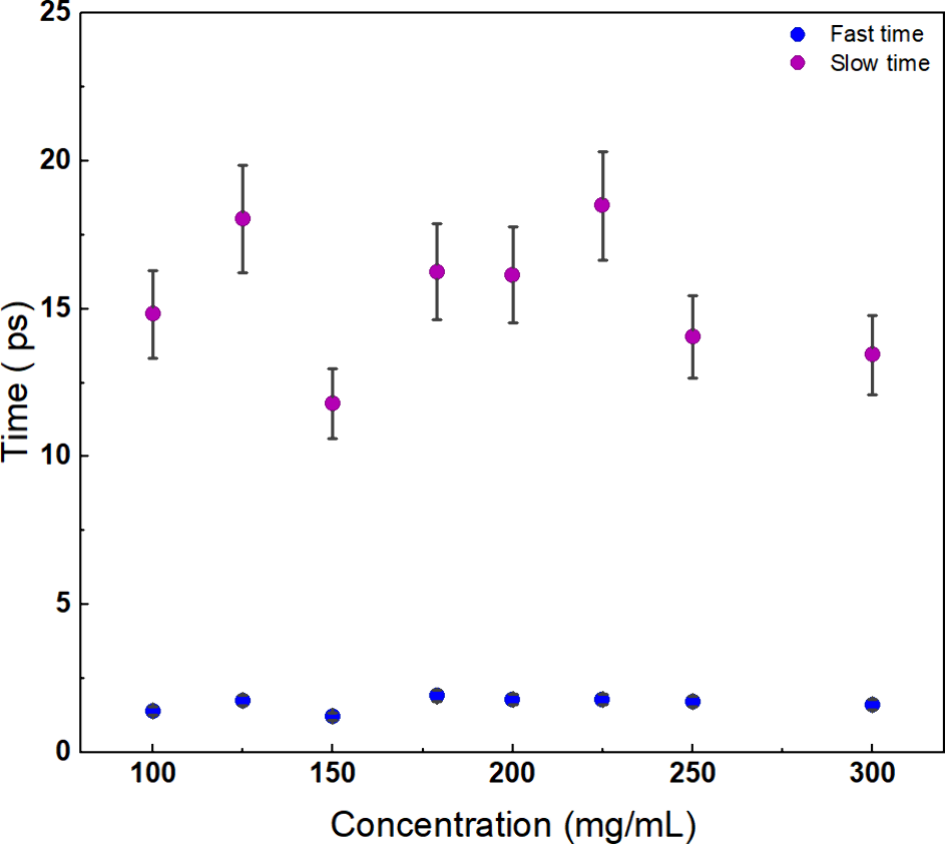
Relaxation times, τ_1_ and τ_2_, extracted from the biexponential fit function reported in section S3.2 of the Supporting Information. The OKE data reveal two distinct relaxation dynamics with well-separated structural relaxation times, though no significant variation with concentration changes is evident within experimental error. However, the OKE data suggest that likely these two dynamics are only weakly influenced by increasing lysozyme concentration or protein clustering effects. The data of a 50 mg/mL lysozyme solution do not enable reliable extraction of relaxation dynamics of the hydration water component, so this has not been reported.

In Figure 4A, we present the sum of the amplitudes of both relaxations measured in this HD-OKE investigation as a function of concentration. The amplitudes exhibit a discontinuity at a specific concentration, approximately 200 mg/mL. This concentration threshold is more evident in the amplitudes than in the relaxation times, possibly because the latter are influenced by non-negligible experimental errors.

The analysis of the vibrational dynamics’ region turns out to be quite complex, and it generates a debate about the assignment of the various vibrational components. The challenge arises mainly from the arduous task of differentiating between hydration and bulk water vibrational contributions.

The HB intermolecular vibrational bands of the hydration water occupy the same spectral region as the bulk water bands.^43^ Also, some protein modes are characterized by vibrational frequencies like water modes. As a result, the hydration water contribution to the vibrational spectrum was typically handled as a component of the global solvent spectrum^27^ or was incorporated into the hydrated protein signal.^21,25^

In our experimental investigation, the OKE signal in the subpicosecond time range, already subtracted from the bulk water contribution, is characterized by two main vibrational contributions of hydration water and the protein intramolecular modes, i.e., vibrations of protein structures and librations of lysozyme amino acid side chains.^27^ As already described, the recorded OKE signal in this time interval was completely reproduced by four DHOs (Figure 1D). The DHO n.3 and n.4 can be assigned to lysozyme librations as proved by the comparison with previous Raman spectra recorded on protein crystals.^44,45^ While DHO n.3 can undoubtedly be assigned to lysozyme dynamics,^40,45^ DHO n.4 could also be partially attributed to the hydration water. The inclusion of a small contribution at this frequency to hydration water does not change the conclusions to which our analysis leads, as it would involve only a rescaling of the amplitudes of the contributions. Nevertheless, the attribution of these mode to lysozyme dynamics is confirmed from the concentration behavior of the sum of the integrated areas of these two DHOs, which follows the lysozyme volume fraction quite well over the entire concentration range (see Figure S5). On the contrary, the DHO n.1 and n.2 contributions in the vibrational spectrum can confidently be attributed to the hydration water modes.

Figure 3A shows the hydration water spectra obtained by subtracting the bulk water and the lysozyme oscillator contributions from the full data spectrum of the 250 mg/mL protein solution. The bulk water component is shown (purple line) for comparison. If we further subtract the structural relaxation component (gray shaded curve), we obtain the clear vibrational modes of hydration water (blue lines and circles); these can be reproduced by two oscillators at 55 and 166 cm^–1^, which are DHO n.1 and n.2, respectively, in the fitting function. These oscillators are quite similar to the modes characterizing the hydration water layers of silica nanopores.^43^ Given their similarity to the vibrational dynamics of bulk water, these two modes can be attributed to the intermolecular bending and stretching vibrations of the hydrogen-bonded network in the water shell surrounding the proteins.

**Figure 3:**
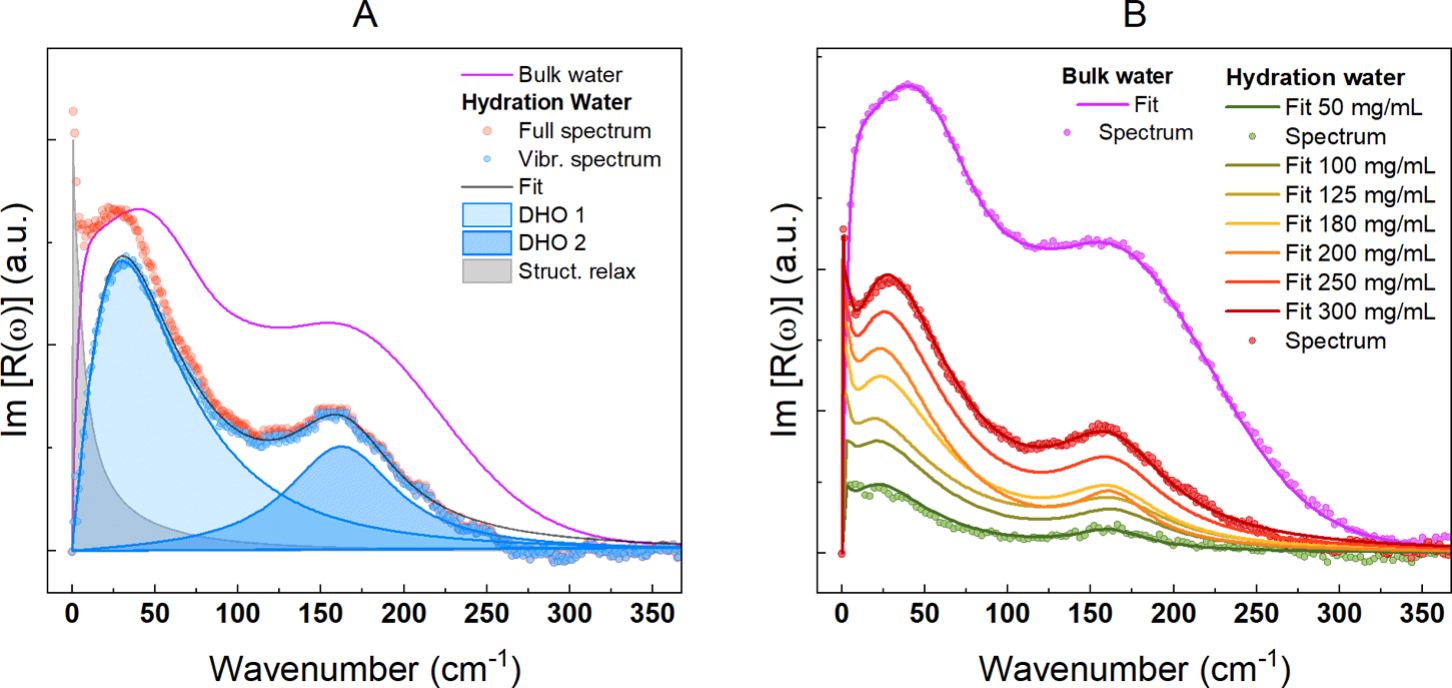
(A) Comparison between the full spectrum (red circles) and the vibrational spectrum (blue circles) of the hydration water dynamics, both extracted from data of the 250 mg/mL lysozyme/water sample; for the sake of completeness, we also show the spectrum of the bulk water component weighted by the bulk water volume fraction (violet line). The fitting result for the structural component (gray shaded line) and the two hydration water oscillators (blue shaded lines, DHO n.1 and DHO n.2) are also reported. (B) Fitting functions and data spectrum of hydration water extrapolated from lysozyme/water data from 50 mg/mL (green line and circles) to 300 mg/mL (red line and circles) and results from a bulk water sample (violet line and circles). We do not report the data of intermediate concentrations for the sake of clarity.

Furthermore, the hydration water spectrum was explored and analyzed in relation to concentration and compared with the bulk water spectrum (see Figure 3B). The central wavenumber of both vibrational modes does not appear to be influenced by the protein crowding effect, as reported in Figure S2. However, the intensity of the modes exhibits a dependence on the protein concentration, revealing an intriguing phenomenon (see Figure 3B). The intensity of the bending mode, DHO n.1, seems to follow an almost monotone trend with an increase in protein concentration. On the contrary, the stretching mode, DHO n.2, follows a different behavior. A strong discontinuity is clearly visible in the hydration water spectral profile, between the yellow (180 mg/mL) and orange (200 mg/mL) curves reported in Figure 3B.

To better emphasize these features, we present in Figure 4B the effective amplitudes of the hydration water vibrational modes, calculated from the integral (the subtended area between 0 and infinite frequency) of the DHO fitting functions versus concentration. Both bending and stretching vibrational amplitudes display a discontinuity around a lysozyme concentration of 200–225 mg/mL, which corresponds to the concentration threshold previously identified in the structural relaxation amplitude analysis. These observations suggest a crossover process in the concentration dependence with the effect being more pronounced for the stretching mode. Before the crossover, both the bending and stretching amplitudes increase steadily with the concentration, driven by the increasing amount of hydration water in the solution. At the crossover, all amplitudes, including the structural one, decrease sharply, indicating a decrease in the hydration water content. Beyond the crossover, the three amplitudes continue to increase, showing a similar increasing rate. These findings suggest the following scenario of protein clustering.

**Figure 4:**
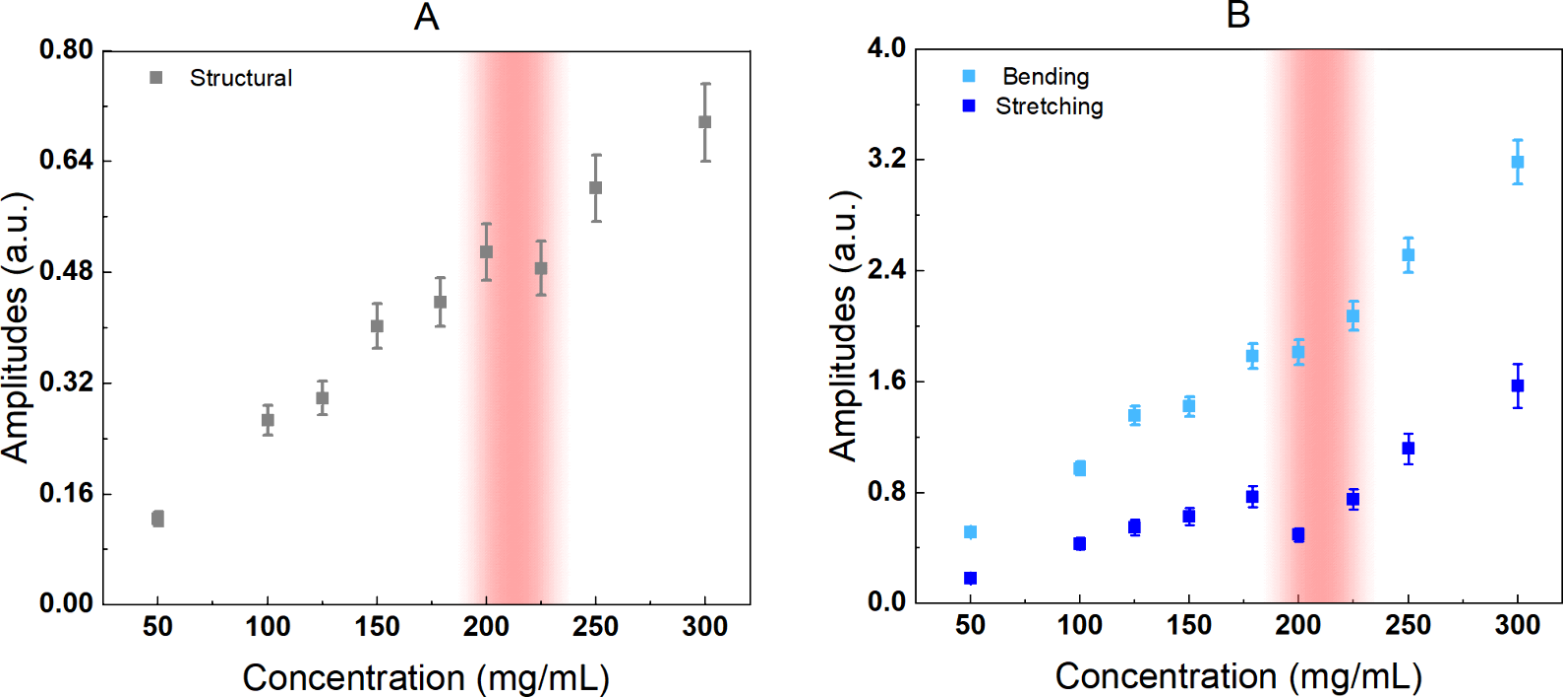
(A) Sum of the amplitudes of the structural relaxations and (B) amplitudes of vibrational modes of the hydration water as a function of concentration. The amplitudes are obtained from the integral areas (we evaluated the subtended area between 0 and infinite frequency) of the different spectral contributions appearing in Figure 3A. Interestingly, all amplitudes show a change around a concentration of around 200–225 mg/mL. This value could indicate the concentration at which lysozyme cluster formation begins to be energetically favored over the condition of closer but unbound single monomers.

Certainly, cluster formation plays a critical role in modulating the hydration water shell. The main effect is the reduction of the protein’s exposed surface area, which consequently alters and reduces the total amount of hydration water. Nonetheless, this effect alone does not fully account for the observed differences between the bending and stretching vibrational modes at higher concentrations. This discrepancy may be attributable to a change in interactions among water molecules at the protein surface, which intensify under high-concentration conditions where clustering is prominent. Such interaction modifications could disproportionately impact the stretching mode, suggesting a distinct sensitivity of this mode to concentration-dependent structural changes at the protein–water interface.

As shown in previous terahertz spectroscopy studies,^17−20^ the occurrence of protein condensation phenomena, induced by the LLPS, modulates the hydration processes. In particular, it reduces the amount of hydration water in the solvation shell close to the protein hydrophobic parts. In these studies, this reduction is evident from the decrease in the amplitude of the vibrational band at 160 cm^–1^ compared to that around 550 cm^–1^, the latter indicating the retention of hydrophilic water. These findings suggest that changes in the population of different types of hydration water, hydrophobic and hydrophilic, can manifest as variations in the amplitudes of the oscillators associated with specific dynamics. The formation of protein clusters, similar to the condensation phenomena, is expected to influence the water solvation shell, especially the first layer of hydration water. The clusters are prone to retaining hydrophilic water while releasing hydrophobic water into the bulk.

This mechanism could explain the crossover effects observed in the amplitudes of the stretching and bending modes, as well as the more pronounced effects on the stretching mode with respect to the bending one. In fact, the hydrophilic water retained within the clusters is expected to hinder the stretching mode compared to the bending mode, as the former requires a larger network to establish compared to the latter.

Our investigations suggest that the clustering phenomena have only a minimal impact on relaxation times and vibrational mode frequencies. While clustering alters the balance between hydrophilic and hydrophobic water within the solvation shell, it appears to have only a minimal effect on the dynamic properties of these two types of hydration water.

In summary, in this HD-OKE investigation, thanks to the high signal-to-noise ratio of our data, precise data analysis and fitting procedures, and an extensive concentration study, we successfully differentiate and quantify the various dynamic processes taking place in a protein/water solution. The focus of our work was on the dynamics of hydration water. We conducted measurements of structural dynamics over extended time windows, encompassing the subpicosecond regime. Our findings revealed the existence of two relaxation processes: one associated with water α-relaxation and another linked to structural reorganization of water coupled with protein fluctuations. This observation, to the best of our knowledge, constitutes the first experimental validation of phenomena predicted by simulation studies^28,29^ and provides crucial insights into the debated understanding of protein hydration water dynamics.

Additionally, our measurement methodology facilitates a direct examination of the vibrational dynamics within the protein solution, allowing for the isolation of hydration water components from overarching signals. Our findings reveal a resemblance between the vibrational spectrum of hydration water and that of bulk water, both exhibiting bending and stretching intermolecular modes. However, distinctive features are observed in hydration water modes, differentiating them from bulk water vibrational modes.

The remarkable capability of the HD-OKE experiment to discern the impacts of exceedingly subtle biomolecular interactions within a solution is noteworthy. Such interactions are notably challenging to detect experimentally by using alternative modes. These findings underscore the potency of the HD-OKE technique as a formidable tool for identifying weak interactions among proteins through the analysis of the hydration water properties.

The investigation of the structural and vibrational dynamics of hydration water components across varying protein concentrations has revealed several intriguing phenomena. The HD-OKE data revealed a specific concentration value, about 200–225 mg/mL, marking a crossover between two distinct clustering regimes identified by discontinuity in the structural relaxation and vibrational mode amplitudes. Our interpretation suggests that this crossover point arises from the effect of the sharp increase in the frequency of protein clustering phenomena as the solution enters a crowded region. The cluster formation modifies the protein solvation shell, reducing the total amount of hydration water and, more specifically, the hydrophobic hydration water with respect to the hydrophilic one. A comparison with the phase diagram of the lysozyme/water solution hints at a correlation between the observed crossover point and the critical point of the LLPS.^14,15^ This connection is further substantiated by the close relationship between the formation of protein clusters and LLPS. Although the temperature at which our measurements are conducted is significantly far from the critical region of LLPS, the observed crossover point seems to suggest an influence of this critical phenomenon on cluster formation within the equilibrium phase of the protein solution.

## Supporting Information Available

Detailed information regarding methods and materials, modeling of the HD-OKE response function, fitting procedure, spectral analysis of the oscillatory components, analysis using two layers of hydration water, volume fraction evaluation, details of the normalization procedure with the water/glycol mixture, and the trend of lysozyme oscillators with concentration (PDF)
