# Supplementary material for: Protein Crowding Effects on Hydration Water Dynamics
Source: J Phys Chem Lett. 2025 Feb 24;16(9):2340–7. doi: 10.1021/acs.jpclett.4c03391 (PMC11891887; doi:10.1021/acs.jpclett.4c03391)
Supplement: Supplementary file 1 — jz4c03391_si_001.pdf [file jz4c03391_si_001.pdf]

Supporting Information for “Protein crowding effects on ultrafast dynamics of hydration water”

Luigi Caminiti[1,2], Maria Taddei[1,2], Sara Catalini[2,3,4], Paolo Bartolini[2], Andrea Taschin[2,4] and Renato Torre[1,2,4*]*

1 Dipartimento di Fisica ed Astronomia, Università degli Studi di Firenze, Via G. Sansone 1, 50019 Sesto Fiorentino (FI), Italy

2 European Laboratory for Non-Linear Spectroscopy, Via Nello Carrara 1, 50019 Sesto Fiorentino (FI), Italy

3 Dipartimento di Fisica e Geologia, Università degli Studi di Perugia, Via Alessandro Pascoli, 06123 Perugia (PG), Italy

4 Consiglio Nazionale delle Ricerche - Istituto Nazionale di Ottica, Via Nello Carrara 1, 50019 Sesto Fiorentino (FI), Italy

Luigi Caminiti: caminiti@lens.unifi.it

Renato Torre: torre@lens.unifi.it

S1. Methods and Materials

S1.1. HD-OKE Set-up

The apparatus used for the HD-OKE measurements is based on a self-mode-locked Ti:sapphire laser (Femtolasers, model Fusion) producing pulses of 15 fs duration, 80 MHz repetition rate and 790 nm wavelength. The experimental set-up details are reported in A. Taschin et al.[1].

S1.2. Sample Preparation

The HD-OKE measurements are performed on lysozyme water solutions at nine different concentrations (50, 100, 125, 150, 180, 200, 225, 250 and 300 mg/mL) at a controlled temperature of 298.0±0.1 K. The samples

were prepared by dissolving the specified amount of dry powder of Hen Egg-White lysozyme (HEWL) obtained from Sigma Aldrich (L6876) in 1 mL of pure water (S.A.L.F. sterile and non-pyrogenic water for injections). We achieved the complete dissolution using Vortex mixing. All the samples were then filtered with a syringe filter (membrane 0.22 μm pore size) to avoid the introduction of dust. We checked the pH value of each sample with a pH Meter (istek 730P) to be around 4.8 as expected by the absence of any added acidic solution. The features of our samples ensure that the globular native shape of the protein is preserved, avoiding the formation of stable aggregates during measurements[2].
For the data normalization procedure, we prepared three solutions of lysozyme (100, 200 and 300 mg/mL) dispersed in a specific mixture of pure water and Glycerol (Sigma Aldrich G7893). The mixture of the two solvents was at 10.74 % of glycerol molar fraction.
All the samples are analysed inside a fused silica cuvette with a dimension of 2x10x30 mm^3.

S2. Modelling of the HD-OKE response function

We present here a simple phenomenological model of the sample dynamics in order to define an analytical expression of the response function needed to fit the HD-OKE signal. The experimental signal recorded can be expressed in the following form[1, 3, 4]:

$$S(t) = \int \left[I_0\delta(t-t') + R(t-t')\right]G(t')dt' \qquad (S2.1)$$

The response function is directly related to the time derivative of the correlation function of the anisotropic first order susceptibility of the sample[1], in the classical approximation we have:

$$R(t) = -\frac{\theta(t)}{k_BT}\frac{\partial}{\partial t}C(t) \qquad (S2.2)$$

$$C(t) = \langle\chi(t)\chi(0)\rangle \qquad (S2.3)$$

where $\theta(t)$ is the Heaviside function, $\chi(t)$ is the off-diagonal component of the collective electronic susceptibility and k_B is the Boltzmann constant. The nuclear response function obtained from the signal can be considered composed of different contributions referring to an operative model that extrapolates from the total function the dynamic observables. In this case, we consider that the total signal is separated in the bulk contribution (R_{bulk}) and the hydration protein contribution (R_{h-Ly}) and, to reproduce the signal with a fitting function, we consider the nuclear response of the hydrated protein as composed by a structural and an oscillating contribution:

$$R_{h-Ly}(t) = R_{struct}(t) + R_{osc}(t) \qquad (S2.4)$$

The first term is reproduced by the time derivative of a bi-exponential decay:

$$R_{struct}(t) = \frac{A_{exp1}}{\tau_1}e^{-\frac{t}{\tau_1}} + \frac{A_{exp2}}{\tau_2}e^{-\frac{t}{\tau_2}} \propto -\theta(t)\frac{\partial}{\partial t}\sum_{i=1}^{2}Exp(t;\tau_i) \qquad (S2.5)$$

The second term can be described by a series of local vibrational modes $Q_n(t)$ that represent an acceptable description of the dynamics of molecular liquids of different nature[4] and that are related to the electronic susceptibility as[1] :

$$\chi(t) \propto \sum_n A_nQ_n(t) \qquad (S2.7)$$

This oscillating model is denoted as *multi-Damped Harmonic Oscillator* (m-DHO)[1, 3], with the coordinates of the $Q_n(t)$ modes following the DHO equation of motion:

$$\ddot{Q}_n(t) + \gamma_n\dot{Q}_n(t) + \omega_n^2Q_n(t) = 0 \qquad (S2.8)$$

The general solution for each single DHO mode is[5]:

$$Q_n(t) = e^{-\frac{\gamma_n}{2}t}(B_ne^{i\Omega_nt} + D_ne^{-i\Omega_nt}) \qquad (S2.9)$$

where $\Omega_n = \sqrt{\omega_n^2 - \frac{\gamma_n^2}{4}}$ and the B_n and D_n constants are defined by the initial conditions.

The combination of these modes must be reported in the time derivative of the autocorrelation function ($C_{osc}(t)$) of the susceptibility for the oscillating contribution (equation S 4.5). Considering the relative boundary conditions[6]:

$$C_{osc}(t) = \sum_n f_{DHO}(t;\Omega_n,\gamma_n) = \sum_n A_n \frac{\left(\frac{\gamma_n}{2} + i\Omega_n\right)}{+i2\Omega_n} e^{-\frac{\gamma_n}{2}t} \left[e^{i\Omega_n t} + \frac{\left(\frac{\gamma_n}{2} - i\Omega_n\right)}{\left(\frac{\gamma_n}{2} + i\Omega_n\right)} e^{-i\Omega_n t} \right] \quad (S2.10)$$

The final function expressed by the oscillating response function for the hydrated protein contribution can be so expressed as:

$$R_{osc} \propto -\theta(t) \frac{\partial}{\partial t} \sum_n f_{DHO}(t;\Omega_n,\gamma_n) = \theta(t) \sum_n \frac{A_n}{\Omega_n} e^{-\frac{\gamma_n t}{2}} sin(\Omega_n t) \quad (S2.11)$$

S3. Fitting Procedure

S3.1. Instrumental function

A very reliable technique is to perform a measurement on a reference sample of calcium fluoride plate (CaF2) placed inside the sample cuvette. The very simple nuclear response of calcium fluoride allows an accurate extraction of the instrumental function[1]. Unfortunately, this approach is not feasible due to the limited size of our cuvette. In Calvagna et al.[3] it's demonstrated that it's possible to build an artificial instrumental function by fitting the first part of the electronic peak (the rise of the signal) with an analytic function that, together with its symmetric image, gives a simulated instrumental function. The result of this method is not accurate as that one obtained with the CaF2 procedure but at the same time it's the better compromise when the best option it's not applicable. So, we decided to extract $G(t)$ using this method.

S3.2. Fitting functions

The fitting functions appearing in the equation (3) of the main text are simulated by few analytic functions[3]. The structural relaxation, $R_{struct}(t)$, is defined by two exponential decays combined with a very fast rising contribution with a fixed parameter is $\tau_R = 50$ fs. This parameter ensures that the response function has its origin at zero.

$$R_{struct}(t) = \frac{A_{exp1}}{\tau_1}\left(1 - e^{-\frac{t}{\tau_R}}\right)e^{-\frac{t}{\tau_1}} + \frac{A_{exp2}}{\tau_2}\left(1 - e^{-\frac{t}{\tau_R}}\right)e^{-\frac{t}{\tau_2}} \quad (S3.1)$$

In the equation *S3.1* we consider a "rise term" $\left(1 - e^{-\frac{t}{\tau_R}}\right)$ to fit the experimental signal. This term is necessary to describe the real growth of the signal which differs from a Heaviside function pure behaviour.

The vibrational response, $R_{osc}(t)$, is described by the time derivative of a series of Damped Harmonic Oscillators (DHO):

$$R_{osc}(t) = \sum_{n=1}^{4} \frac{A_n}{\sqrt{\omega_n^2 - \frac{\gamma_n^2}{4}}} e^{-\frac{(\gamma_n t)}{2}} sin\left(\sqrt{\omega_n^2 - \frac{\gamma_n^2}{4}}\, t\right) \quad (S3.2)$$

The errors associated with the fitting parameter are primarily influenced by the exact reproducibility of the experimental conditions and measurements. As a result, these errors are estimated by comparing the dispersion of values from numerous measurements of the same sample.

S3.3. Selection of the numbers of DHOs

A careful observation of the present HD-OKE data reveals that the time evolution exhibits a complex temporal structure characterized by overlapping and beating oscillatory components extending across a wide range of frequencies. Using the response function outlined in the previous equation, we are forced to limit the number of Damped Harmonic Oscillators (DHOs) in the analysis.

In Figure S1, we present a comparison between two fitting procedures: one using three DHOs and the other using four DHOs. The fit with three DHOs successfully captures most of the dynamics but fails to reproduce a specific oscillation occurring in the 0.5–1 ps range. In contrast, the four-DHO fit accurately reproduces the

entire dynamics, particularly by incorporating an additional oscillator with a frequency of approximately 110 cm^{-1}.
However, neither fit can capture the finer structures in the data, which are characterized by very rapid oscillations. Accurately reproducing these features would require adding multiple high-frequency DHOs, significantly complicating the fitting process. Consequently, we fixed the number of DHOs at four, as this represents the minimum required to adequately reproduce the main dynamic features.
All fits were performed directly in the time domain, which proved highly sensitive in revealing specific features of the dynamics. On the other hand, the frequency domain was particularly useful for identifying the frequencies of individual oscillators.

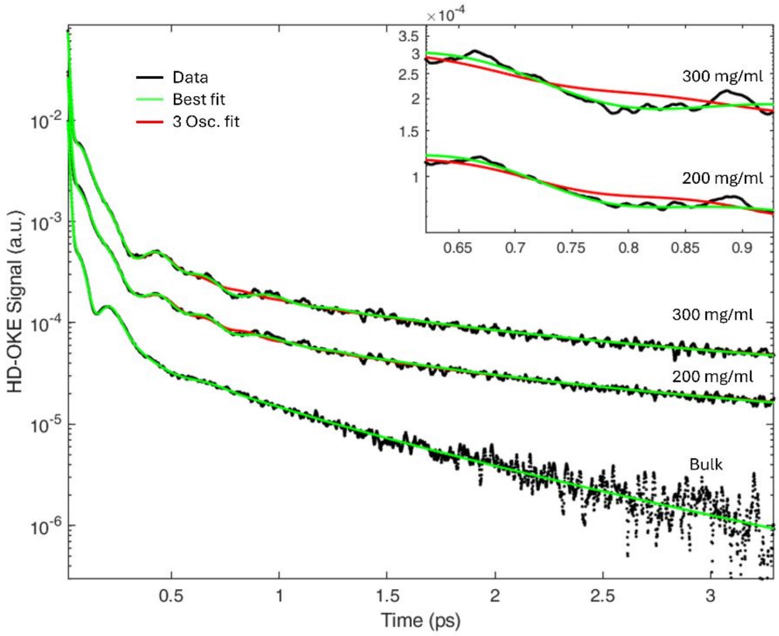

Fig. S1. Fit-data comparison for concentrations of 200 mg/ml and 300 mg/ml is shown in a linear-log plot. Each curve is scaled by an appropriate factor to avoid overlapping. Experimental data (black lines) are reproduced using the model described in equation 3 of the main text, where the R_{osc} component of R_{h-ly} is modeled as the sum of four (green lines) or three (red lines) oscillators. For comparison, bulk water data and its corresponding fit are also reported. The inset highlights that the oscillation around 0.8 ps is accurately reproduced only when four oscillators are included.

S4. Spectral analysis of the oscillatory components

In Figure S2 we report the frequencies associated with the four dumped Lorentzian oscillators as a function of the concentration of lysozyme in solution. All the frequencies obtained from the fits don't show a strong dependence on the increase in concentration. This result allows us to state that there are no evident variations in the vibrational dynamics of the samples as a function of the concentration in conditions of constant pH and temperature.

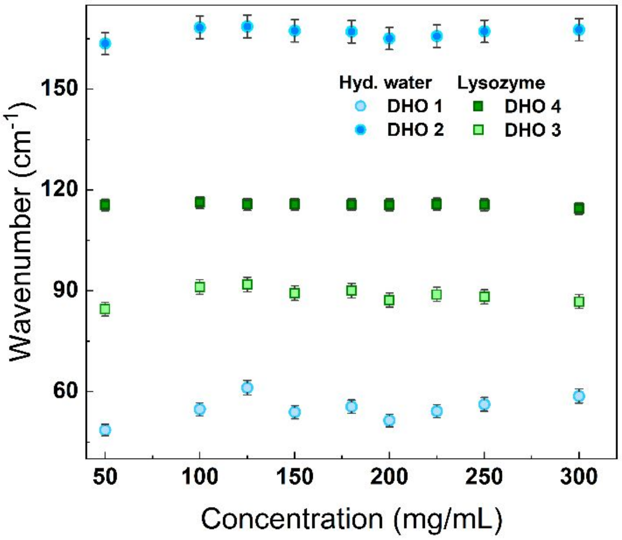

Fig. S2. Central oscillation frequency of the damped Lorentzian functions used to fit the vibrational contribution of the hydrated protein ($\boldsymbol{R_{h-Ly}}$) for each Lysozyme solution. The green squares are associated to the lysozyme vibrational components, while the blue circles to hydration water vibrational components. Each value is reported with an error bar evaluated comparing the dispersion of values of numerous measurements of a same sample.

S5. Analysis using two layers of hydration water

The analysis described in the main text was replicated using a hydration water shell consisting of two layers of hydration water. The number of water molecules forming the two layers is 1076 for each lysozyme molecule. This value is taken from Camisasca et al.[7]. This second analysis simply shows a rescaling of all the amplitudes of the fitting function (equation 3 of the main text), maintaining the same concentration trend shown in the main text using the analysis with only one layer of hydration water. A comparison of the oscillators assigned to hydration water obtained using the one-layer (dashed line) and two-layer (dotted line) analyses is shown in Fig. S3. As can be seen, the oscillators have the same shape, differing only by a scaling factor.

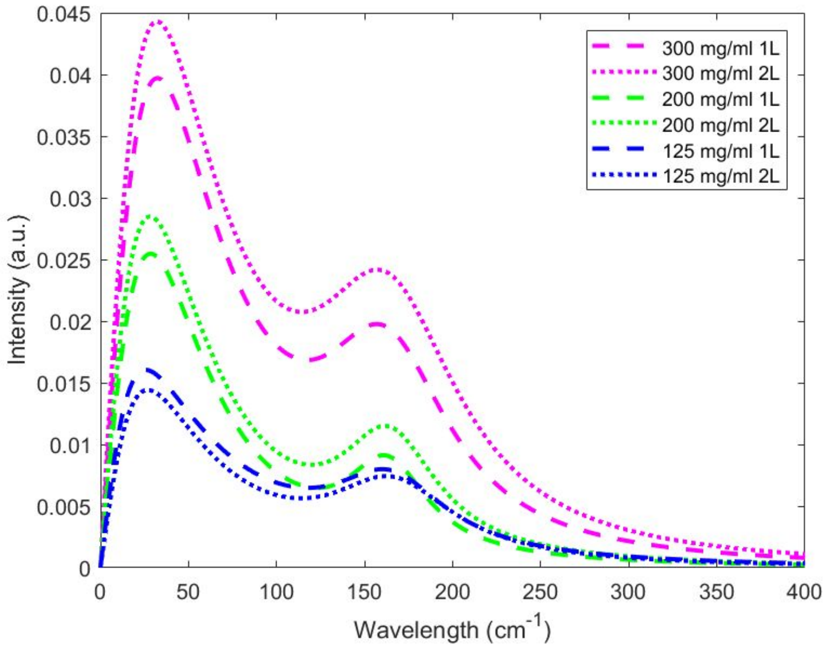

Fig. S3. Comparison between the one-layer (dashed line) and two-layer (dotted line) analyses. In the figure are shown the fitting function of the two oscillators assigned to the hydration water contribute. The results are taken from the measures of the samples at the concentration of 125 mg/ml (blue lines), 200 mg/ml (green lines) and 300 mg/ml (magenta lines). The results from the two analyses don't show trend differences using one or two layers of hydration water. The two analyses give a scale factor difference.

S6. Volume fraction evaluation

The molar fraction of bulk water in solution at a certain protein concentration depends on the number of water molecules considered in the hydration layer around the single biomolecules. In this work, we consider lysozyme as an ellipsoid with semi-axis dimensions of 2.25×1.5×1.5 nm[8], and we evaluate the number of water molecules around its volume following the characterization in *G. Camicasca et al.*[7]. They report a Radial Distribution Function (RDF) study made on lysozyme solutions where the first layer of water molecules appears to be restrained within a radius of 4 Å from each protein surface. Inside this layer, at 300 K they calculate 604 water molecules. Therefore, we referred to this first layer as the hydration water component around lysozyme, while all the residual water was considered as bulk water. In Table 1, we report the values of all the volume fractions calculated for bulk water, hydration water, and lysozyme contribution.

Table 1. Values of the volume fraction calculated for bulk water, hydration water, and lysozyme in all the samples analysed. They are calculated following the hydration model reported in the Method section.

	Water	*50 mg/mL*	*100 mg/mL*	*125 mg/mL*	*150 mg/mL*	*180 mg/mL*	*200 mg/mL*	*225 mg/mL*	*250 mg/mL*	*300 mg/mL*
Bulk water	1.000	0.922	0.85	0.815	0.783	0.745	0.721	0.692	0.665	0.611
Hydration water	0.000	0.036	0.069	0.085	0.100	0.117	0.128	0.142	0.154	0.179
Lysozyme	0.000	0.042	0.081	0.100	0.117	0.138	0.151	0.166	0.181	0.210

S7. Details of the normalization procedure with the water-glycerol mixture

To compare the response function of each sample at different lysozyme concentrations, we implemented a normalization procedure. Several previous studies normalize data based on the electronic peak. However, this method is flawed because it fails to account for the impact of concentration on the intensity of this peak. The HD-OKE signal recorded for an aqueous lysozyme solution does not exhibit distinct bands for either the solute or the solvent within the frequency range typically accessible by these measurements. Thus, direct normalization cannot be achieved. We decided to conduct a series of additional HD-OKE measurements on the water-protein solutions by introducing an external species with a well-characterized and distinct signal relative to the lysozyme-water sample. This approach allows us to directly account for the intensity effect of concentration.

We added glycerol as a 10.74% molar fraction of water solvent. Glycerol was chosen because of its low influence on the protein-water system[9] and it generates contribution to the signal characterized by an intense oscillating pattern with a short period on the time scale. We acquired the HD-OKE signal at three concentrations of lysozyme in water-glycerol (100, 200 and 300 mg/mL). Our procedure is based on these data and a series of steps of rescaling of the data. The first step of this procedure corresponds to the standard normalization in the time domain on the electronic peak. Next, we transitioned the data analysis to the frequency domain: the HD-OKE signals was Fourier transformed, and the instrumental function was deconvoluted in the frequency domain. In the frequency domain we can select a spectral region in which only glycerol vibrational bands are present: 750-1010 cm^{-1}. We evaluate the integral area of these bands, the value obtained is proportional to the volume fraction of glycerol in solution and hence of the water-glycerol volume fraction. We have therefore normalised the HD-OKE signals for the water-glycerol-lysozyme samples so that the integral of the selected glycerol bands decreases proportionally with the decrease in glycerol volume fraction as the lysozyme concentration increases (see Fig. S4). We can now establish a calibration line. To do this, we return to the time domain and evaluate the integrals of the signals in this domain. Plotting the values obtained as a function of the lysozyme concentrations and using the integral of the water-glycerol signal at 0 mg/ml lysozyme as a unitary reference value, we perform a linear fitting procedure to obtain the coefficients of the calibration line. We can assume that the signals obtained from the samples without glycerol also follow this calibration law. Finally, we normalised all nine HD-OKE (water-lysozyme) sample signals so that their time integral follows the calculated calibration line.

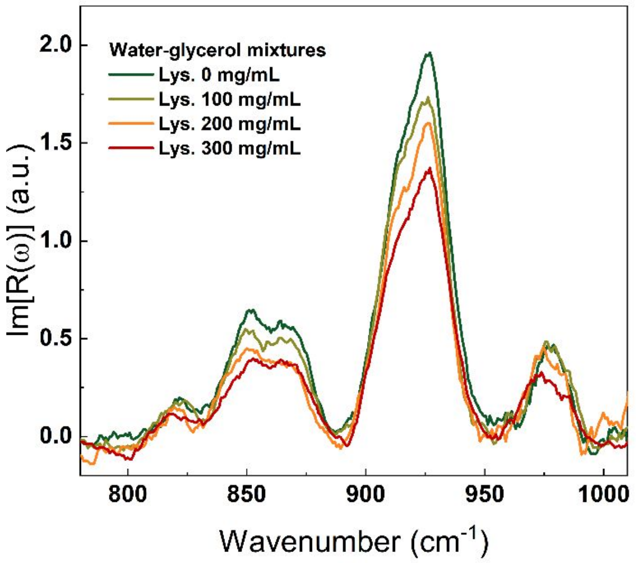

Fig. S4. HD-OKE signal at three concentrations of lysozyme in the mixture water-glycerol (100, 200 and 300 mg/mL), normalized in the time domain on the electronic peak.

S7. Lysozyme oscillators trend with concentration

Figure S5 show the behaviour of the sum of the amplitudes of the two DHOs assigned to lysozyme. the attribution of these mode at lysozyme dynamics is confirmed from the concentration behaviour of the sum of the integrated areas of the these to DHOs, which follows the lysozyme volume fraction quite well over the entire concentration range.

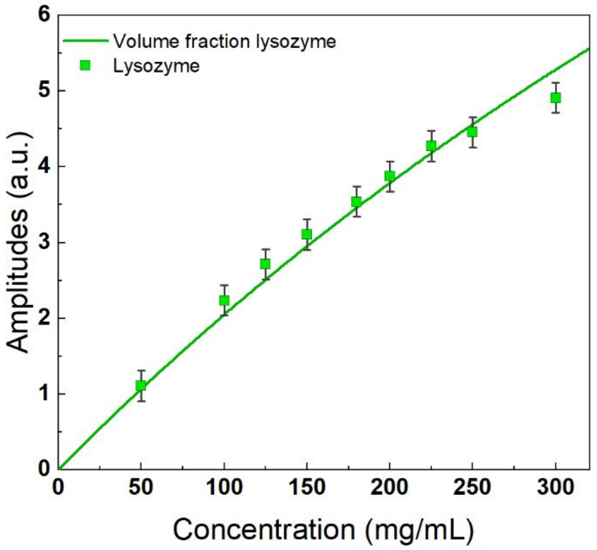

Fig. S5. Sum of the amplitudes of the two DHOs assigned to lysozyme internal mode and the expected trend evaluated from the volume fraction that the protein occupies in the sample at different concentrations.

References

(1) Taschin, A.; Bartolini, P.; Eramo, R.; Righini, R.; Torre, R. Optical Kerr effect of liquid and supercooled water: The experimental and data analysis perspective. *The Journal of chemical physics* **2014**, *141* (8), 084507-084507. DOI: 10.1063/1.4893557.
(2) Miti, T.; Mulaj, M.; Schmit, J. D.; Muschol, M. Stable, metastable, and kinetically trapped amyloid aggregate phases. *Biomacromolecules* **2015**, *16* (1), 326-335. DOI: 10.1021/bm501521r.
(3) Calvagna, C.; Lapini, A.; Taschin, A.; Fanetti, S.; Pagliai, M.; Bartolini, P.; Bini, R.; Righini, R.; Torre, R. Modification of local and collective dynamics of water in perchlorate solution, induced by pressure and concentration. *Journal of Molecular Liquids* **2021**, *337*, 116273. DOI: 10.1016/j.molliq.2021.116273.
(4) Bartolini, P.; Taschin, A.; Eramo, R.; Torre, R. Optical Kerr Effect Experiments on Complex Liquids, a Direct Access to Fast Dynamic Processes. In *Time-Resolved Spectroscopy in Complex Liquids*, Torre, R. Ed.; Springer US, 2008; pp 73-127.
(5) Morin, D. *Introduction to classical mechanics : with problems and solutions*; Cambridge University Press, 2008.
(6) Bafile, U.; Guarini, E.; Barocchi, F. Collective acoustic modes as renormalized damped oscillators: Unified description of neutron and x-ray scattering data from classical fluids (vol 73, pg 061203, 2006). *Physical Review E* **2006**, *74* (5). DOI: 10.1103/PhysRevE.74.059902.
(7) Camisasca, G.; Iorio, A.; De Marzio, M.; Gallo, P. Structure and slow dynamics of protein hydration water. *Journal of Molecular Liquids* **2018**, *268*, 903-910. DOI: 10.1016/j.molliq.2018.07.104.
(8) Blake, C. C.; Grace, D. E.; Johnson, L. N.; Perkins, S. J.; Phillips, D. C.; Cassels, R.; Dobson, C. M.; Poulsen, F. M.; Williams, R. J. Physical and chemical properties of lysozyme. *Ciba Foundation symposium* **1977**, (60), 137-185. DOI: 10.1002/9780470720424.ch10 From NLM.

(9) Sinibaldi, R.; Ortore, M. G.; Spinozzi, F.; Carsughi, F.; Frielinghaus, H.; Cinelli, S.; Onori, G.; Mariani, P. Preferential hydration of lysozyme in water/glycerol mixtures: A small-angle neutron scattering study. *The Journal of chemical physics* **2007**, *126* (23), 235101. DOI: 10.1063/1.2735620.
